# Naked1 Antagonizes Wnt Signaling by Preventing Nuclear Accumulation of β-Catenin

**DOI:** 10.1371/journal.pone.0018650

**Published:** 2011-04-07

**Authors:** Terence J. Van Raay, Nicholas J. Fortino, Bryan W. Miller, Haiting Ma, Garnet Lau, Cunxi Li, Jeffery L. Franklin, Liliana Attisano, Lilianna Solnica-Krezel, Robert J. Coffey

**Affiliations:** 1 Department of Medicine, Vanderbilt University Medical Center, Nashville, Tennessee, United States of America; 2 Department of Biological Sciences, Vanderbilt University, Nashville, Tennessee, United States of America; 3 Department of Molecular and Cellular Biology, University of Guelph, Guelph, Ontario, Canada; 4 Department of Biochemistry and Donnelly Centre for Cellular and Biomolecular Research, University of Toronto, Toronto, Ontario, Canada; 5 Department of Cell and Developmental Biology, Vanderbilt University Medical Center, Nashville, Tennessee, United States of America; 6 Department of Pediatrics, Vanderbilt University Medical Center, Nashville, Tennessee, United States of America; 7 Department of Veterans Affairs Medical Center, Nashville, Tennessee, United States of America; University of Oldenburg, Germany

## Abstract

Cyto-nuclear shuttling of β-catenin is at the epicenter of the canonical Wnt pathway and mutations in genes that result in excessive nuclear accumulation of β-catenin are the driving force behind the initiation of many cancers. Recently, Naked Cuticle homolog 1 (Nkd1) has been identified as a Wnt-induced intracellular negative regulator of canonical Wnt signaling. The current model suggests that Nkd1 acts between Disheveled (Dvl) and β-catenin. Here, we employ the zebrafish embryo to characterize the cellular and biochemical role of Nkd1 *in vivo*. We demonstrate that Nkd1 binds to β-catenin and prevents its nuclear accumulation. We also show that this interaction is conserved in mammalian cultured cells. Further, we demonstrate that Nkd1 function is dependent on its interaction with the cell membrane. Given the conserved nature of Nkd1, our results shed light on the negative feedback regulation of Wnt signaling through the Nkd1-mediated negative control of nuclear accumulation of β-catenin.

## Introduction

Wnt signaling is involved in many aspects of development and again in the homeostasis of certain stem cells in the adult, such as those found in hematopoietic, hair follicle and intestinal crypt stem cells [1], [2], [3], [4]. Consequently, dysregulation of Wnt signaling during development or in the adult can cause disease [1], [5], [6]. Under normal conditions, canonical or Wnt/β-catenin signaling is initiated upon Wnt ligands binding to their transmembrane receptors. This activates Disheveled (Dvl), which then inhibits the constitutively active destruction complex consisting of Axin, GSK3β and APC. Inhibition of this destruction complex results in cytoplasmic accumulation of β-catenin, which then translocates into the nucleus to activate its transcriptional program. Two consistent, and perhaps universal targets of Wnt signaling are the negative feedback regulators Naked Cuticle Homolog 1 (Nkd1) and Axin2 [7], [8], [9], [10].

Whereas it has been demonstrated that Axin2 functions similarly to Axin1, by binding to β-catenin and inducing its degradation [11], [12], the mechanism by which Nkds antagonize Wnt signaling is less understood. In vertebrates, there are two Nkd homologues: Nkd1 and Nkd2, which are equally similar to Naked Cuticle sharing approximately 45% amino acid identity and we previously demonstrated that both can inhibit canonical Wnt/β-catenin and non-canonical Wnt/PCP signaling [9]. But there are significant expression and functional differences between the two homologues to strongly suggest that Nkd1 is the Naked Cuticle orthologue. First, *nkd1* expression recapitulates many, if not all, of the known Wnt signaling events during development [9], [10], [13] and is upregulated in cancers that are known to have activating mutations in the Wnt/β-catenin pathway [14], [15]. In contrast, *nkd2* expression does not appear to be under the control of Wnt signaling [9], [13], [16]. Functionally, Nkd2 can regulate Wnt signaling specifically by targeting Dvl1 for degradation [17]. But Nkd2 also has a role in escorting TGFa to the basolateral surface of polarized epithelial cells [18], [19], [20], [21]. In contrast, Nkd1 does not share these functions with Nkd2 [20] and thus far Nkd1 appears specific for Wnt signaling [14], [22].

Previous work in *Drosophila* has established that Nkd acts between Dvl and β-catenin, and its inhibitory activity is dependent on intact and active Wnt signaling [23]. Subsequent work has suggested that Nkd has a nucleo-cytoplasmic role, shuttling either Dvl or other signaling components out of the nucleus [24]. In both *Drosophila* and in mammalian *in vitro* assays, Nkd was found to interact physically with the PDZ domain of Dvl [22], [23] and Dvl binds Nkd/Nkd1 in at least two domains: a conserved region encompassing the EF-hand domain and a region in the C-terminal half of Nkd1 [10], [20], [22]. In *Drosophila*, the EF-Hand of Nkd alone is required for binding to Dvl [25], but curiously, Nkd mutant proteins lacking the EF-Hand are capable of rescuing *nkd*-/- mutants to adulthood [26]. This suggests that in Drosophila the interaction between Nkd and Dvl is dispensable for Nkd activity. Previously we demonstrated that Nkd2 is myristoylated. This modification targets Nkd2 to the plasma membrane and is required for its proper function [20]. While the myristoylation sequence is conserved in Nkd1, it is not conserved in fly Nkd. Nevertheless, fly and mosquito Nkds share a unique N-terminal sequence that confers functional properties and membrane association to Nkd [27].

Further insight into Nkd1 function was recently gained using an integrated physical and functional screen to identify Wnt inhibitors [28]. Using this approach, it was found that Nkd1 interacts with Axin and this interaction is required to antagonize Wnt signaling. In this screen, it was also found that Nkd1 had a significant interaction with β-catenin but it is unknown if this is dependent on Axin or vice versa [28]. These recent findings imply that Nkd1 is not acting solely at the level of Dvl and that its interactions with other components of the Wnt/β-catenin pathway are likely involved in the ability of Nkd1 to inhibit Wnt signaling.

To probe further the functional significance of the Nkd1-β-catenin interaction *in vivo*, we employed the zebrafish model. At dome stage, which is approximately 4.3 hours post fertilization (hpf), the zebrafish blastula contains approximately 4000 to 8000 pluripotent cells that have yet to undergo differentiation or gastrulation [29]. At this stage, the embryo has been undergoing active zygotic transcription for about 1.3 hours [30], but is only initiating the first zygotic canonical Wnt/β-catenin signaling event, via Wnt8, along the ventro-lateral margin [9], [31], [32]. While endogenous canonical Wnt/β-catenin signaling is only occurring along the ventro-lateral margin, the entire blastula at this stage is responsive to canonical Wnt/β-catenin signaling [33], [34]. Furthermore, the non-canonical Wnt/PCP pathway is not yet active at this stage and so we took advantage of these canonical Wnt/β-catenin-responsive cells to delineate the function of Nkd1 in antagonizing canonical Wnt/β-catenin signaling, hereafter referred to as Wnt signaling. Using this system and overexpression assays, we demonstrate that Nkd1 interacts with β-catenin and acts to prevent β-catenin from accumulating within the nucleus. These results provide insight into the negative feedback mechanism of endogenous Wnt signaling in an *in vivo* model, specifically at the level of cyto-nuclear distribution of β-catenin.

## Results

### Myristoylation sequence is required for plasma membrane localization

**Figure 1:**
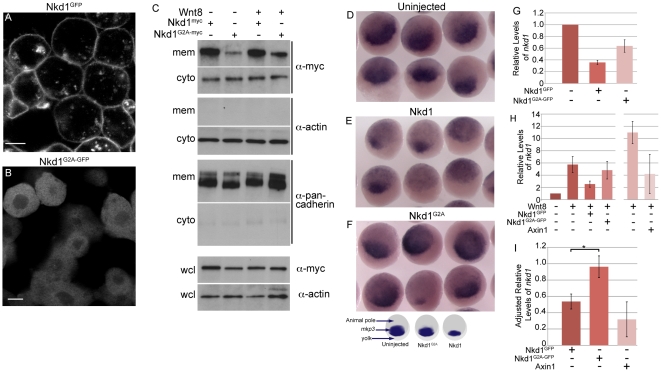
Plasma membrane localized Nkd1 is required for antagonizing Wnt signaling. *nkd1^GFP^* (A) or *nkd1^G2A-GFP^* (B) mRNA was injected into 1 of 4 blastomeres and allowed to develop until dome stage (4.3 hpf). Embryos were viewed live with confocal microscopy. (C) In a similar experiment, embryos injected at the one cell stage with either *nkd1^myc^* or *nkd1^G2A^* ^-*myc*^ in combination with *wnt8,* were collected at dome stage and fractionated into cytoplasmic (cyto) or plasma membrane (mem) fractions. Western blots of fractions were probed with anti-myc. This blot was also probed with anti-actin or anti-pancadherin to determine purity of fractions. Each lane represents the equivalent of 1 embryo from an average of 10 embryos. To determine total amount of protein, a portion of the pre-fractionated whole cell lysate (wcl) was western blotted and probed with anti-myc and anti-actin as a loading control. For the wcl, each lane represents the equivalent of 0.75 of an embryo from an average of 10 embryos. (D, E, F) Embryos were injected at the one cell stage with either *nkd1* (E) or *nkd1^G2A^* (F) RNA and harvested, along with uninjected controls (D), at sphere stage (3.8 hpf). Embryos were processed for whole mount in situ hybridization using a *mkp*3 anti-sense probe [38]. The schematic below (F) summarizes the whole mount in situ hybridization data, identifies the different regions of the embryo and the domain of *mkp3* expression in the dorsal organizer region. (G) Embryos were injected at the 1 cell stage with *nkd1^GFP^* or *nkd1^G2A-GFP^*, harvested at dome stage and total RNA was isolated and reversed transcribed. qRT-PCR was performed using endogenous *nkd1* as readout. The 5′ untranslated region of *nkd1* was used as a target, the sequence of which was not incorporated into the expression vectors. Values are relative to uninjected embryos which was arbitrarily set to 1 (N = 3). (H) Quantification of endogenous *nkd1* levels by qRT-PCR in the presence of Wnt8. (N = 6 for Wnt8; Wnt8+Nkd1^GFP^; Wnt8+Nkd1^G2A-GFP^). The Wnt8+Axin experiments (N = 3) were done independently of the others, which showed higher induction of *nkd1* by Wnt8 alone, compared to the other experiments and relative to uninjected. (I) To adjust for the differences between experiments described in (H), the ratio of endogenous levels of *nkd1* was determined for Wnt8+Nkd1^GFP^:Wnt8 (N = 6), Wnt8+Nkd1^G2A-GFP^:Wnt8 (N = 6) and Wnt8+Axin1:Wnt8 (N = 3) for each individual experiment and then averaged. (* = Students t-test, p = 0.0252). A level of one indicates no effect. Error bars represent standard error. Scale bar in A,B represents 10 µm.

The N-terminal myristoylation sequence is highly conserved between vertebrate Nkd homologues and it has been previously demonstrated that human Nkd2 is myristoylated [9], [20]. Thus, we wanted to determine if zebrafish Nkd1 is plasma membrane bound and if this is myristoylation sequence dependent. Immunohistochemical analysis of mosaically overexpressed Nkd1^GFP^ (C-terminal GFP tag) showed enriched protein expression at the plasma membrane at dome stage (4.3 hpf) (Fig. 1A). In addition, there were also large and small GFP positive puncta within the cytoplasm, reminiscent of Dvl puncta (Fig. 1A) [35], [36]. To confirm that Nkd1 is plasma membrane localized, zebrafish blastula (4.3 hpf) overexpressing Nkd1^myc^ (C-terminal myc tag) alone or with Wnt8 were homogenized and the lysate was fractionated to isolate the plasma membrane and cytoplasmic fractions (Fig. 1C). Consistent with the immunohistochemistry data, we found Nkd1^myc^ to be enriched within the plasma membrane fraction, although some Nkd1^myc^ was also detected in the cytoplasmic fraction (Fig. 1C). Co-injection of *wnt8* RNA did not appear to alter the overall levels of plasma membrane or cytoplasmic Nkd1^myc^. To determine if the above subcellular distribution of Nkd1 was dependent on myristoylation, we mutated the second amino acid, glycine, to alanine (G2A) to generate Nkd1^G2A-GFP^ or Nkd1^G2A-myc^. This mutation abolishes the myristoylation activity of human Nkd2 [20]. In contrast to Nkd1^GFP^, Nkd1^G2A-GFP^ was no longer plasma membrane-enriched and no longer formed the cytoplasmic puncta as assayed by immunohistochemistry (Fig. 1B). Instead, Nkd1^G2A-GFP^ became evenly distributed within the cytoplasm and possibly in the nucleus as well. Fractionation experiments confirmed the loss of plasma membrane association, as there was dramatically less plasma membrane enrichment of Nkd1^G2A-myc^ relative to Nkd1^myc^ (Fig. 1C). The fractionation data also revealed that a portion of Nkd1^G2A-myc^ still segregated with the plasma membrane fraction, which was not obvious by immunohistochemistry (Fig. 1 B,C).

We also noticed that the levels of cytoplasmic plus membrane Nkd1^G2A^ proteins did not add up to the levels detected in the whole cell lysate when compared to wild-type Nkd1 by Western blot analysis. We generated a Nkd1^G2P^ mutant form and observed that it was also unstable in our fractionation experiments (not shown), even though a proline at the N-terminal end of proteins is predicted to be stable [37]. This suggests that the fractionation procedure is unfavorable to Nkd1^G2A/P^ stability. In general, we found that Nkd1 was more stable than Nkd1^G2A^ (TVR submitted) and so more Nkd1^G2A^ RNA was injected to compensate for this difference.

### Nkd1 Myristoylation is required to antagonize Wnt activity

To ascertain if the myristoylation sequence of Nkd1 was required for antagonizing Wnt activity, we overexpressed Nkd1 and Nkd1^G2A^ and assayed for *mkp3* expression by whole mount in situ hybridization at sphere stage (3.8. hpf). At this stage, *mkp3* is a direct target of the maternal Wnt/β-catenin pathway [38] but shortly after becomes regulated by FGF signaling [38]. We observed that Nkd1^G2A^ had very little effect on *mkp3* expression in contrast to Nkd1 overexpression, which led to a reduction in *mkp3* expression (Fig. 1 D–F). This was confirmed using quantitative RT-PCR (qRT-PCR). Total RNA from embryos injected with either *nkd1^GFP^* or *nkd1^G2A-GFP^* RNA was assayed for endogenous *nkd1* expression at dome stage (4.3 hpf) as readout of active Wnt signaling (Fig. 1G). Consistent with the whole mount in situ hybridization data, we observed that Nkd1^GFP^ could reduce the expression of a Wnt target gene, while a myristoylation-deficient form of Nkd1 (Nkd1^G2A^) was less effective at doing so.

To determine if Nkd1^G2A^ could prevent the activation of target genes induced by excess Wnt signaling, we performed qRT-PCR on embryos co-injected with *wnt8* and *nkd1^GFP^* or *nkd1^G2A-GFP^* RNAs. For comparison we also co-injected *wnt8* and *axin1* RNA, the latter encoding a potent inhibitor of Wnt signaling (Fig. 1H,I). We observed that Nkd1^GFP^ and Axin1 reduced the effect of ectopic Wnt8 on endogenous *nkd1* expression, with Nkd1^GFP^ having slightly less influence than Axin1. In contrast, Nkd1^G2A-GFP^ was markedly less active in this assay (Fig. 1H,I). Therefore, we conclude that myristoylation of Nkd1 is required for its ability to antagonize Wnt signaling, suggesting that plasma membrane association of Nkd1 is critical for its activity. The latter conclusion is similar to results found in *Drosophila* where removal of the N-terminal sequence of Nkd abrogated both its plasma membrane localization and its ability to function. This N-terminal-deleted Nkd isoform was also much less stable [27]. This argues for conservation of function, as both fly and vertebrate Nkd/Nkd1 appear to require association with the plasma membrane for proper activity in antagonizing Wnt signaling.

### Nkd1 binding to Dvl2 is independent of myristoylation

**Figure 2:**
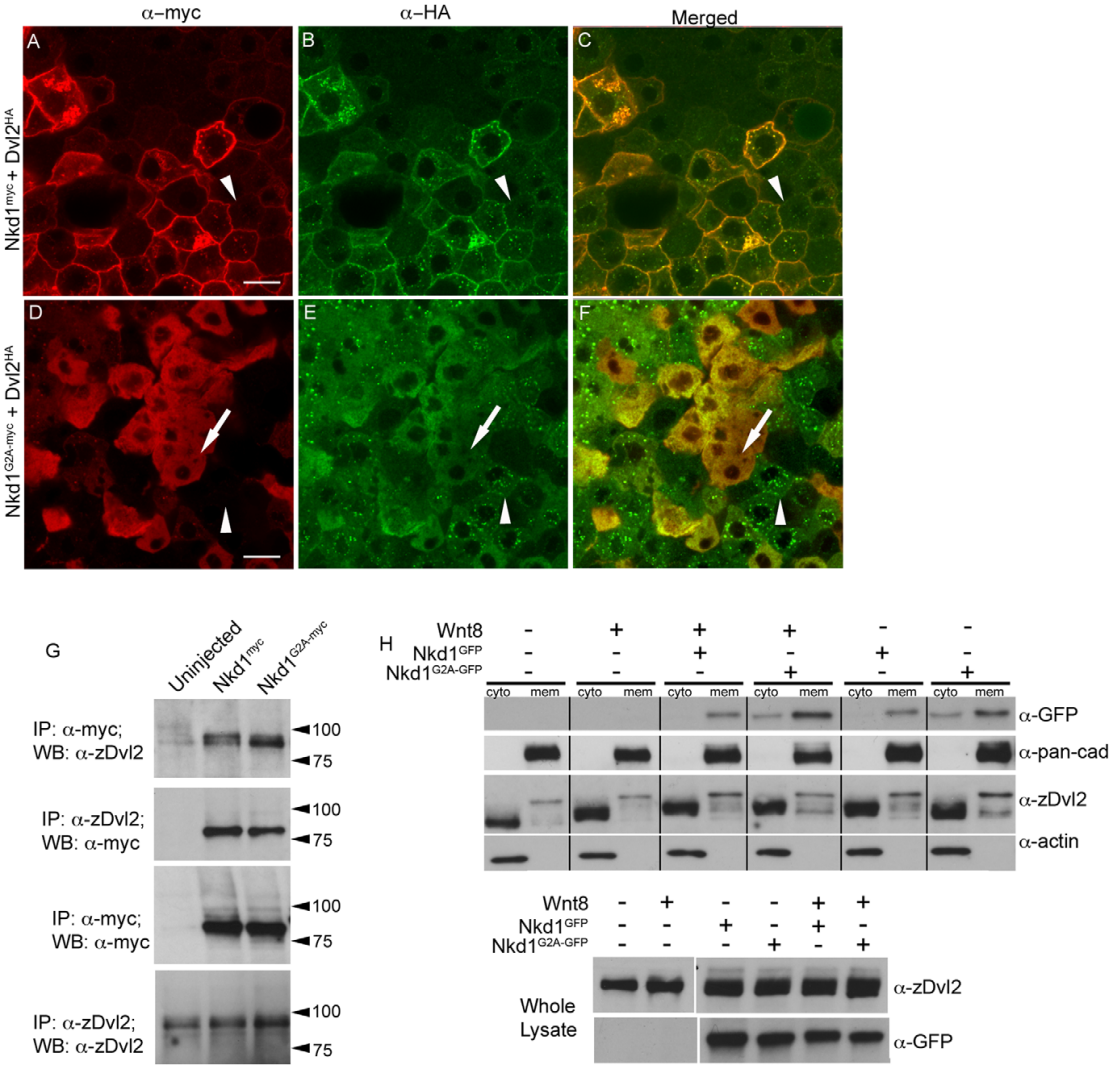
Nkd1 co-localizes with Dvl2. (A–F) *Dvl2^HA^* mRNA was injected at the one cell stage followed by injection of *nkd1^myc^* or *nkd1^G2A-myc^* RNA into 1 of 4 blastomeres at the 4-cell stage. Embryos were harvested at 50% epiboly (5.25 hpf) and processed for immunohistochemistry using anti-myc probes (red) or anti-HA probes (green). Arrowheads in A-F identify a cell that is positive for Dvl2^HA^ but negative for Nkd1^myc^ (A–C) or Nkd1^G2A-myc^ (D–F). Arrow in D–F indentifies a cell that is positive for both Dvl2^HA^ and Nkd1^G2A-myc^. (G–H) Nkd1 interacts with Dvl2. (G) Embryos were injected at the one cell stage with *nkd1^myc^* or *nkd1^G2A-myc^* and harvested at dome stage (4.3 hpf) for co-immunoprecipations. Lysates were incubated with anti-zDvl2 or anti-myc antibodies for immunoprecipitation. Immunoprecipitates were western blotted and probed with anti-zDvl2 or anti-myc antibodies (WB). Each lane represents the equivalent of 17.5 embryos. (H) *nkd1^GFP^* or *nkd1^G2A-GFP^* RNAs were co-injected with *wnt8* RNA at the one cell stage. At 4.3 hpf, embryos were harvested and fractionated to isolate cytoplasmic (cyto) and plasma membrane (mem) fractions. Anti-actin and anti-pan-cadherin antibodies determined the purity of the cytoplasmic and plasma membrane fractions, respectively. A portion of the whole cell lysate was recovered prior to fractionation (lower panel) to determine loading controls. (Note: the high percentage gel precluded observing Dvl mobility shifts induced by Wnt8 in the whole cell lysate). The predicted MW of Nkd1^myc^ is 61 kD, but the observed MW is closer to 75 kD. We speculate that this is likely due to post-translational modifications of Nkd1. Each lane represents the equivalent of 1 embryo, averaged from 10 embryos. Scale bar represents 20 µm.

Previous studies have demonstrated that Nkds bind Dvl proteins [10], [22], [23], [39]. Therefore, we wanted to determine if Dvl binding was dependent on Nkd1 myristoylation. Immunohistochemistry of blastula (4.3 hpf) overexpressing Nkd1^myc^ and Dvl2^HA^ in a mosaic fashion showed that Dvl2^HA^ co-localized with Nkd1^myc^ at the plasma membrane but also in cytoplasmic puncta (Fig. 2A–C). It is possible that Nkd1^myc^ stabilized Dvl2^HA^ at the plasma membrane, as adjacent cells expressing Dvl2^HA^ alone exhibited low plasma membrane staining (arrowheads in Fig. 2A–C). In contrast, immunohistochemistry with Dvl2^HA^ and Nkd1^G2A-myc^ demonstrated that cells co-expressing both Dvl2^HA^ and Nkd1^G2A-myc^, but not Dvl2^HA^ alone, have lost the Dvl2^HA^ puncta staining (compare arrows and arrowheads, Fig. 2D–F), arguing that Nkd1^G2A-myc^ can still bind to Dvl2^HA^ and dissociate Dvl2^HA^ puncta.

To confirm that Nkd1^myc^ interacts with endogenous Dvl2 and to determine if Nkd1^G2A-myc^ can also bind to endogenous Dvl2, we performed co-immunoprecipitation assays. Consistent with the immunohistochemistry data, we observed that Nkd1 could interact with Dvl2 independent of its myristoylation, but the molecular weights of Dsh^HA^ were different between Nkd1^myc^ and Nkd1^G2A-myc^ (Fig. 2G). We observed Nkd1^myc^ immunoprecipitated with Dvl2 isoforms that migrate with different mobilities, when compared to those complexed with Nkd1^G2A-myc^ (Fig. 2G). Nkd1^GFP^ interacted with both slow and fast migrating isoforms of Dvl2, while Nkd1^G2A-GFP^ interacted preferentially with the fast migrating isoform. To pursue the effect of Nkd1 on Dvl2 further, we co-injected RNAs encoding *nkd1^GFP^* or *nkd1^G2A^* ^-*GFP*^ with *wnt8* RNA and fractionated the embryo lysates to observe the plasma membrane and cytoplasmic forms of Dvl2. In the cytoplasmic fractions, Dvl2 undergoes a characteristic mobility shift due to phosphorylation in the presence of Wnt8 [40], [41], [42], [43], which was also observed for the two Nkd1 constructs (Fig. 2H). In agreement with the co-immunoprecipitation experiment, we also observed different banding patterns in the plasma membrane fractions of Dvl2 between Nkd1^GFP^ and Nkd1^G2A-GFP^, which appear independent of the addition of Wnt8 (Fig. 2H). Specifically, we observed an increase in intensity of the slower and intermediate migrating bands in the presence of Nkd1^GFP^, but an increase in a fast migrating band of Dvl2 in the presence of Nkd1^G2A-GFP^ when compared to uninjected or *wnt8* RNA alone injected embryos (Fig. 2H).

The differences in plasma membrane vs cytoplasmic levels of Dvl2 observed by western blot analysis did not exactly correlate with the immunohistochemical data (Fig. 2) as we did not see an increase in endogenous Dvl2 in the membrane fraction by western, while we observed increased membrane localization with exogenous Dvl2^HA^ by immunohistochemistry. This may be due to fundamental differences between ectopic and endogenous Dvl2, or that membrane association of Dvl2 is lost during the fractionation process. Nonetheless and taken together, this data suggests that Nkd1^GFP^ modifies, interacts with or stabilizes different isoforms of Dvl2. While Nkd1^G2A-GFP^ also interacts with Dvl2, it is doing so differently than Nkd1^GFP^. This is consistent with the ability of Nkd1, but not Nkd1^G2A^, to inhibit ectopic Wnt8 signaling (Fig. 1I).

### Nkd1 does not alter cytoplasmic levels of β-catenin

**Figure 3:**
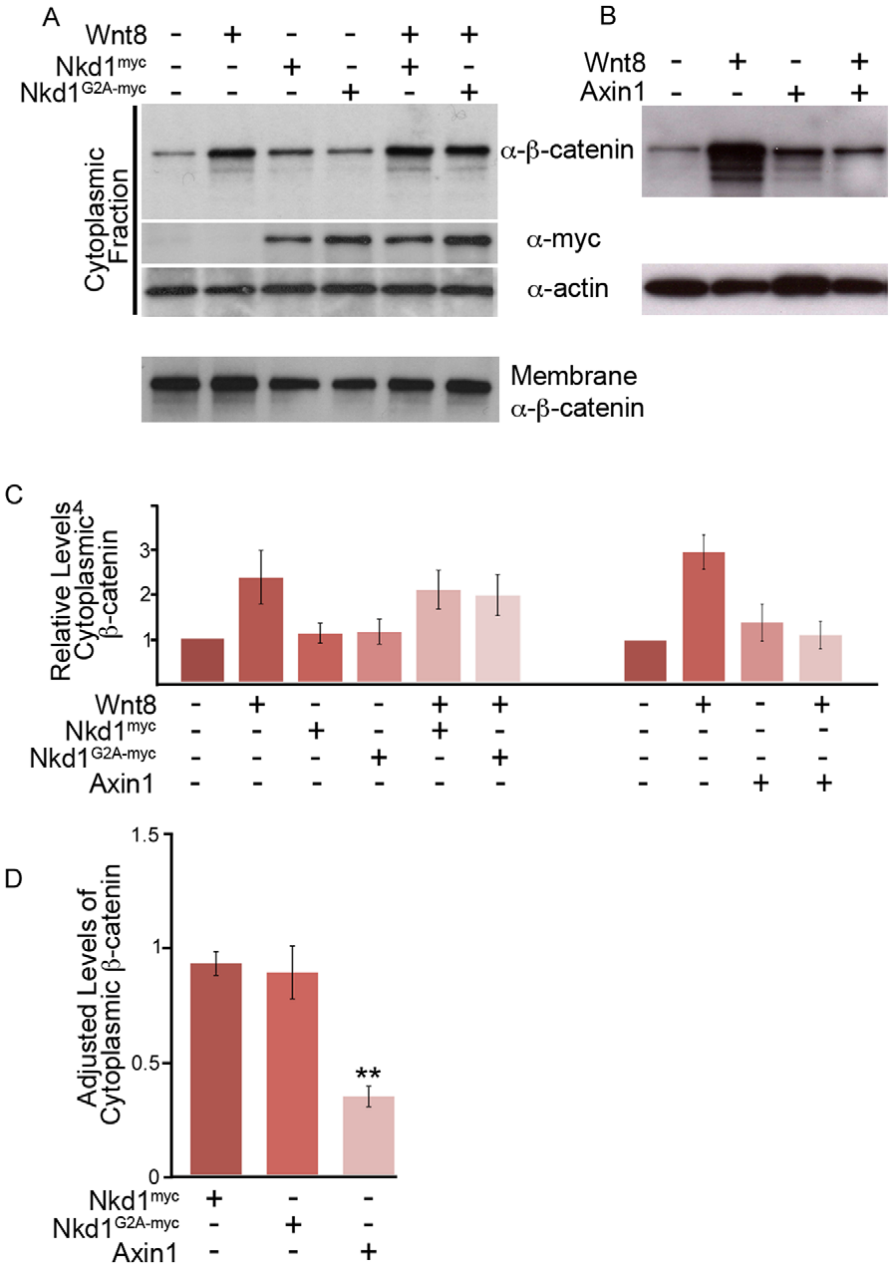
Nkd1 does not affect cytoplasmic levels of β-catenin. (A,B) Embryos were injected at the one stage with *wnt8*, *nkd1^myc^* or *nkd1^G2A^* ^-*myc*^ (A) or *axin1* (B) and harvested at dome stage (4.3 hpf). Lysates were fractionated into plasma membrane (not shown) and cytosolic fractions, Western blotted and probed with anti-β-catenin, anti-myc and anti-actin antibodies. Each lane represents, on average, the equivalent of one embryo averaged from 10 embryos. (C) The average levels of cytosolic β-catenin from 6 independent experiments (Wnt8+/- Nkd1^myc^/Nkd1^G2A-myc^) or from 3 independent experiments (Wnt8+/-Axin1) were determined. Each lane was first normalized to actin levels and then compared to uninjected embryos, which was arbitrarily set to 1. D) To adjust for the differences between experiments described in (C), the ratio of cytoplasmic levels of β-catenin was determined for Wnt8+Nkd1^myc^:Wnt8 (N = 6), Wnt8+Nkd1^G2A-myc^:Wnt8 (N = 6) and Wnt8+Axin1:Wnt8 (N = 3) for each individual experiment and then averaged. (**-Students t-test, p = 0.0002). A level of one indicates no effect. Error bars represent standard error.

Thus far, we have determined that plasma membrane localization of Nkd1 is required for its activity to antagonize Wnt signaling. We have also demonstrated that Nkd1 binds to and somehow modifies, interacts with, or stabilizes different Dvl2 isoforms. These specific interactions and modifications also appear to occur at the plasma membrane. A model based on genetic epistasis studies suggests that Nkd1 acts between Dvl and β-catenin [23], with the simplest and most attractive model being that Nkd1 acts at the level of Dvl, somehow binding to and inactivating Dvl [10], [23], [24], [26], [27], [44]. If Nkd1 is indeed antagonizing Wnt signaling at the level of Dvl, then Nkd1 should prevent the accumulation of cytoplasmic β-catenin induced by Wnt8. To test this model, we co-injected *nkd1* or *nkd1^G2A^* RNAs (either with myc or GFP tags) with or without *wnt8* RNA, isolated the cytoplasmic fraction and assayed for β-catenin by Western blot (Fig. 3A). Inconsistent with the model described above, we observed no decrease in cytoplasmic β-catenin levels in the presence of Nkd1 or Nkd1^G2A^ with or without Wnt8 (Fig. 3A,C,D). To determine that this is qualitatively different than Axin, we next compared the effect of Nkd1 to Axin1. Consistent with the known function of Axin1, we observed reduced cytoplasmic levels of β-catenin induced by Wnt8 in the presence of Axin1 (Fig. 3B,D). These experiments were repeated 6 times with similar results (Fig. 3C; Table 1). Taken together, we conclude that while the constitutively active Axin1 destruction complex destabilizes cytoplasmic β-catenin, Nkd1 does not appear to antagonize the Wnt pathway at this level, arguing that Nkd1 may instead be acting downstream of stabilized cytoplasmic β-catenin.

**Table 1:** Relative cytoplasmic levels of β-catenin.

Injection	Average	Ttest p-values
Uninj	1.0	
Wnt8^1^	2.4	0.05 †
Nkd1^myc^	1.1	0.61 †
Nkd1^G2A-myc^	1.2	0.60††
Wnt8+Nkd1^myc^	2.1	0.72††
Wnt8+Nkd1^G2A-myc^	2.0	0.61††
Wnt8^2^	3.2	0.02 †
Axin1	1.4	0.38 †
Wnt8+Axin1	1.1	0.04††

Averages were taken from 6 (Nkd1^myc^ or Nkd1^G2A-myc^ co-injections) or 3 (Axin1 co-injections) independent experiments and first normalized to actin levels. Uninjected levels were set to 1.00 and other levels are relative to uninjected.

1 Nkd1^myc^ and Nkd1^G2A-myc^ co-injection series.

2 Axin1 co-injection series.

† -Students t-test (2 tailed, equal variance) compared to uninjected;

†† -Students t-test (2 tailed, equal variance) compared to Wnt8 injected.

### Nkd1 prevents Wnt8-induced nuclear accumulation of β-catenin

**Figure 4:**
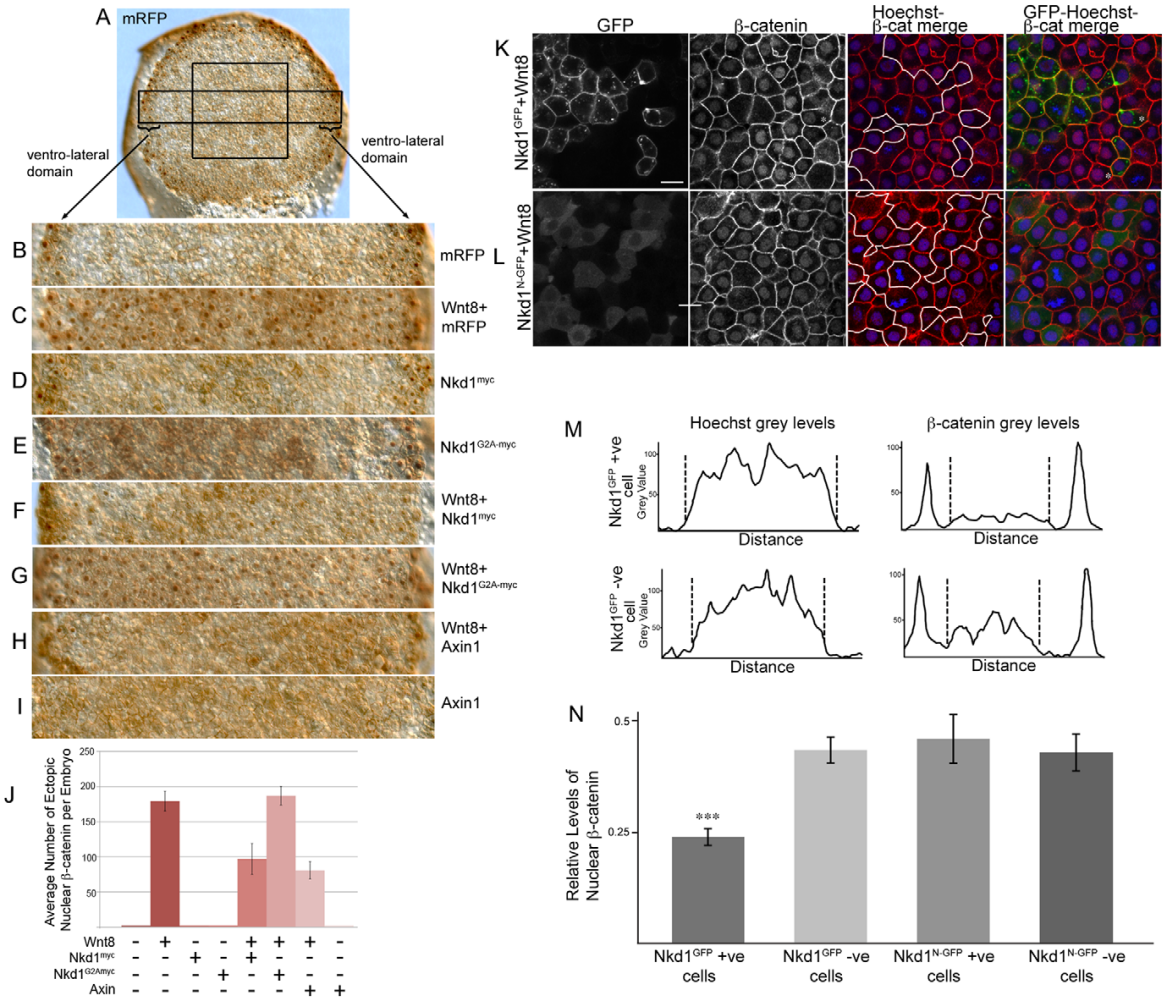
Nkd1 inhibits accumulation of nuclear β-catenin. (A–I) Embryos were injected at the one cell stage with *mRFP* (A,B); *wnt8+mRFP* (C); *nkd1^myc^* (D); *nkd1^G2A^* ^-*myc*^ (E); *wnt8*+*nkd1^myc^* (F); *wnt8*+*nkd1^G2A^* ^-*myc*^ (G); *wnt8*+*axin1* (H); and *axin1* alone (I). At dome stage (4.3 hpf) embryos were collected and processed for whole-mount immunohistochemistry with anti-β-catenin. The expression of nuclear β-catenin around the ventro-lateral margin of the embryo is due to endogenous Wnt8 activity. Note that Axin1 is also sufficient to prevent nuclear localization of β-catenin induced by endogenous Wnt8 activity. The box in (A) represents the size and position of analysis for (J). The rectangle in (A) depicts the regions shown in (B–I) which encompasses the ventro-lateral margins on either side of the embryo. (J) Blind counts of the number of cells expressing ectopic nuclear β-catenin was quantified (Table 2). Error bars represent standard error. To confirm the effect of Nkd1 on nuclear β-catenin, *wnt8* RNA was co-injected with *nkd1^GFP^* (K) or *nkd1^N^* ^-*GFP*^ RNA (L) into 1 of 4 blastomeres, harvested at dome stage and processed for endogenous β-catenin staining. Regions of mosaic expression were chosen for analysis. Clones of Nkd1^GFP^ or Nkd1^G2A-GFP^ positive clones are outlined in white. Black and white images of GFP and β-catenin are shown for contrast. See also Fig. S1 for the complete set of images. (M-N) Quantification of the differences in nuclear β-catenin from six cells (Wnt8+Nkd1^GFP^) or 10 cells (Wnt8+Nkd1^N-GFP^) cells each for GFP positive/Hoechst positive and juxtaposed GFP negative/Hoechst positive cells. The graphs in (M) represents the two cells identified by asterisks in the right hand-most image of (K). The dashed lines delineate the nuclear grey levels. (N) The level of nuclear β-catenin was adjusted using the Hoechst staining. Only the grey values located between the dashed lines were evaluated. (p value  = 0.0002). Error bars represent std error. Scale bar represents 20 µM.

As Nkd1 does not significantly alter the levels of cytoplasmic β-catenin, we wanted to determine if Nkd1 altered the levels of nuclear β-catenin. To test this we overexpressed Wnt8 with or without Nkd1^myc^ or Nkd1^G2A-myc^ and performed whole mount immunohistochemistry against β-catenin at dome stage (4.3 hpf). GFP mock-injected embryos displayed nuclear β-catenin around the perimeter of the embryo as a result of endogenous ventro-lateral Wnt8 activity (Fig. 4A,B) [32]. However, there was a complete absence of nuclear β-catenin in cells at the animal pole at this stage (Fig. 4A,B,J). Injection of *wnt8* RNA alone induced nuclear β-catenin in the majority of cells in the animal pole region at this stage (Fig. 4C,J). Injection of *nkd1^myc^* or *nkd1^G2A-myc^* RNA alone had only a modest effect on ventro-lateral nuclear β-catenin, consistent with our previous observations (Fig. 4D,E,J) [9]. In contrast, injection of *axin1* RNA resulted in significantly fewer ventro-lateral cells with nuclear β-catenin (Fig. 4J). When RNAs encoding *wnt8* and *nkd1^myc^* were co-injected, there was a substantial reduction in both ectopic and ventro-lateral nuclear β-catenin (Figs. 4F,J). In contrast, ectopic Nkd1^G2A-myc^ was unable to prevent the accumulation of nuclear β-catenin induced by Wnt8 overexpression (Fig. 4G,J). This data was consistent across numerous embryos and experiments (Fig. 4J and Table 2). Based on these results, we suggest that Nkd1 functions upstream of nuclear accumulation of β-catenin and potentially functions by preventing nuclear accumulation of β-catenin.

**Table 2:** Nuclear β-catenin counts.

Injection	Ave # cells with Nuclear β-catenin	number of embryos	Ttest p-values*
mGFP	2.00	6	
Wnt8	179.22	9	
Nkd1^myc^	2.00	6	
Nkd1^G2A-myc^	2.00	6	
Wnt8+Nkd1^myc^	97.00	6	0.002794
Wnt8+Nkd1^G2A-myc^	187.00	10	0.346332
Wnt8+Axin	80.67	6	0.000144
Axin	2.00	6	

Nuclear β-catenin counts from the central square shown in figure 4. * Students t-test (2 tailed, equal variance) compared to Wnt8 injected.

To test further the hypothesis that Nkd1 functions at the level of nuclear β-catenin accumulation, we co-injected RNAs encoding *wnt8* and *nkd1^GFP^* or *wnt8* and *nkd1^N^* ^-*GFP*^ (an N-terminal GFP fusion and more stable form of myristoylation deficient Nkd1) into 1 of 4 blastomeres at the 4-cell stage. We reasoned that if Nkd1^GFP^ somehow affects nuclear β-catenin, we would predict GFP positive cells to have reduced levels of nuclear β-catenin, compared to juxtaposed, GFP negative cells, which would have elevated levels of nuclear β-catenin due to the non-cell autonomous effects of Wnt8. Accordingly, we observed a decrease in nuclear β-catenin in Nkd1^GFP^ positive cells, compared to juxtaposed and surrounding cells that were GFP negative (Fig. 4K,M,N). As the nucleus of every cell is not in the same plane of focus, we stained the nuclei with Hoechst, a general DNA marker, and adjusted the level of β-catenin staining intensity according the Hoechst staining intensity (Fig. 4M, S1). The Hoechst staining confirmed that nuclei in the Nkd1^GFP^ positive cells were visible and that they had significantly less β-catenin staining when compared to juxtaposed GFP negative cells (Fig. 4N, S1).

In contrast, Nkd1^N-GFP^ was much less efficient at reducing the levels of nuclear β-catenin (Fig. 4L,N). We conclude that myristoylated Nkd1 likely acts downstream of stabilized cytoplasmic β-catenin, preventing its nuclear accumulation.

### Nkd1 physically interacts with β-catenin

**Figure 5:**
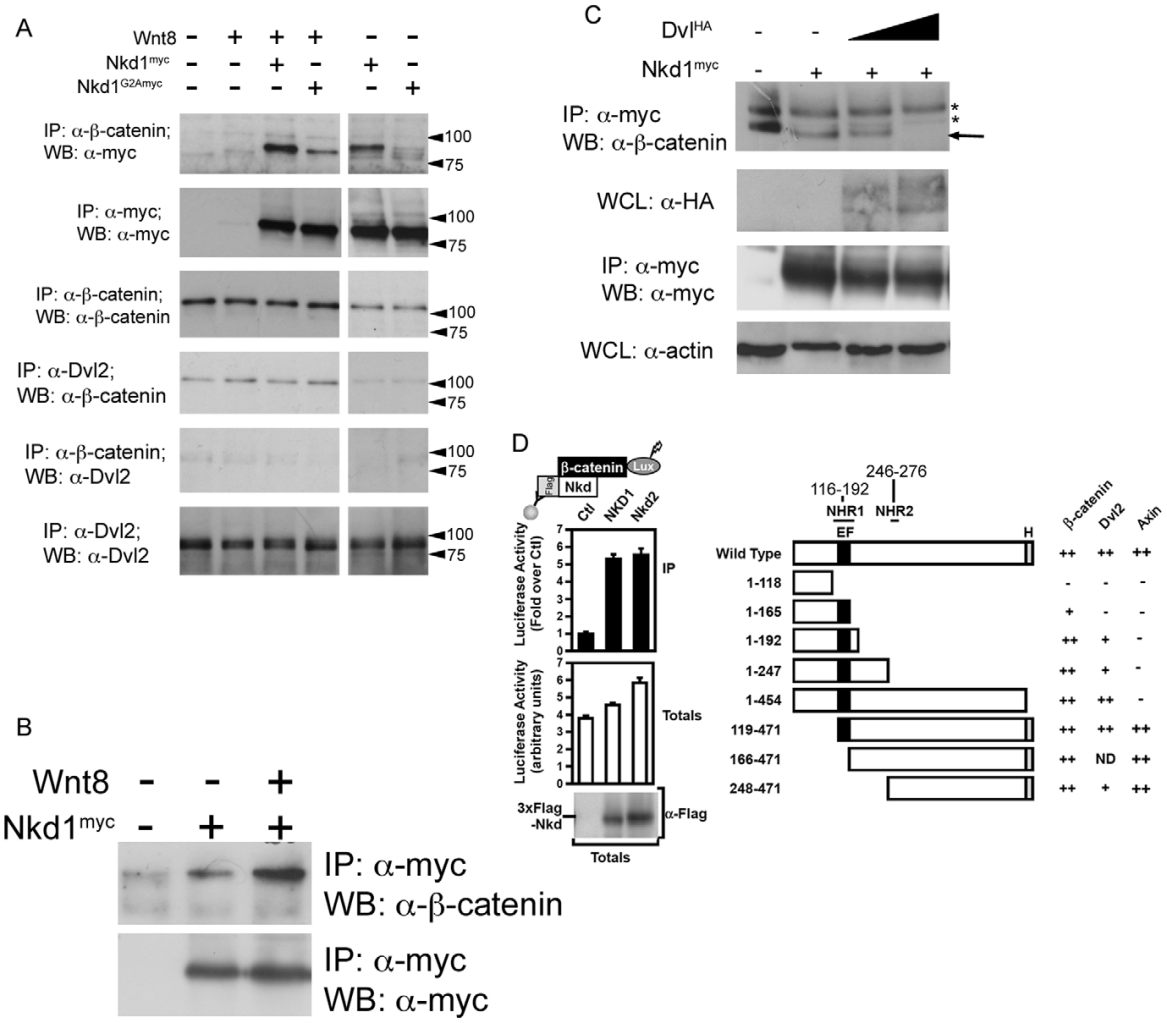
Nkd1 interacts with β-catenin. To determine if Nkd1 and β-catenin interact, *wnt8* +/− *nkd1^myc^* or *nkd1^G2A^* ^-*myc*^ was injected at the 1 cell stage and harvested at dome stage for immunoprecipitation with anti-β-catenin (A). Each lane represents the equivalent of 17.5 embryos. Note, this experiment was part of a larger experiment involving the co-immunoprecipitations shown in Fig. 2 (B) and as such share some of the same controls. (B) *nkd1^myc^* +/− *wnt8* was injected into 1 cell stage embyros and harvested at dome stage, immunoprecipitated with anti-myc and western blotted with anti-β-catenin. The interaction between Nkd1 and β-catenin is much stronger in the presence of exongenous Wnt. Slight non-specific binding of the anti-myc antibody is observed in the control lane. A no primary antibody control showed no banding pattern (not shown). C) *nkd1^myc^* was co-injected with increasing doses of *dsh^HA^* (0pg, 125pg and 250pg) harvested at dome stage, immunoprecipitated with anti-myc and western blotted with anti-β-catenin. The astericks identify non-specific banding patterns. D) The interaction of luciferase-tagged β-catenin (β-catenin-Lux) with wild type Nkd1 and Nkd2 (left panel) and Nkd1 mutants (right panel) was tested using the LUMIER assay in HEK293T cells. Numbers above Wild Type Nkd1 refer to amino acid positions in human Nkd1. Axin data has been previously reported [28] and is shown here for completeness. Data are shown as the mean of 2 samples +/− standard deviation (left panel) and interaction intensities of less than 50% (−), 50–75% (+) and >75% (++) of wild type are indicated (right panel). clarity we refer to NHR1 as Naked Homology Region 1 (116–192aa), which encompasses the EF hand (143–154aa). The NHR1 region has also been referred to as NH2 [56] as well as EFX [10].

β-catenin is at the center of the canonical Wnt signaling cascade and the nucleo-cytoplasmic shuttling of this protein is the focus of recent research [45], [46]. We found that while Nkd1 is enriched at the plasma membrane with cytoplasmic puncta, it does not appear to alter cytoplasmic levels of β-catenin (Fig. 3A). Instead our studies suggest that Nkd1 prevents the nuclear accumulation of β-catenin. Therefore, we wanted to determine if there was a physical interaction between Nkd1^myc^ and β-catenin. By co-immunoprecipitation, we observed a strong interaction between Nkd1^myc^ and β-catenin, but a much weaker interaction between Nkd1^G2A-myc^ and β-catenin (Fig. 5A). This suggests that Nkd1 myristoylation and its association with the plasma membrane is important for binding to β-catenin, although we do not know if this a direct or indirect interaction. We also observed an increase in Nkd1-β-catenin interaction in the presence of Wnt8 (Fig. 5B); however, it is unclear if this is a result of increased levels of cytoplasmic β-catenin due to excess Wnt8, or if Wnt signaling alters the affinity of Nkd1 for β-catenin, or both. Furthermore, these experiments cannot distinguish which pool of β-catenin (plasma membrane or cytoplasmic) interacts with which pool of Nkd1 (plasma membrane or cytoplasmic puncta).

Since Nkd1 also binds to Dvl2, we were curious if we could observe a ternary complex between Nkd1, Dvl2 and β-catenin. Immunoprecipitating Dvl2 we detected a low background level of co-immunoprecipitated β-catenin that did not change in the presence of Wnt, Nkd1^myc^ or Nkd1^G2A-myc^, whereas the reverse IP suggested little or no interaction (Fig. 5A). Even though Nkd1 was found to interact with both Dvl2 and β-catenin (Figs. 2G, 5A), this suggests that Dvl2, Nkd1 and β-catenin may not co-exist in a ternary complex and further that Dvl2 and β-catenin may compete for binding to Nkd1. To test this, we co-injected increasing amounts of Dvl2^HA^ and observed a dramatic reduction in the levels of β-catenin that immunoprecipitated with Nkd1^myc^ with increasing amounts of Dvl2^HA^ (Fig. 5C).

Using human Nkd1 deletion mutants [28], we mapped the regions of Nkd1 that are required to bind β-catenin. We found that β-catenin binds to Nkd1 in a conserved region encompassing the EF-hand (NHR1 domain), as well as in a region C-terminal to this, which includes a second region of conserved homology (NHR2) [9]. Further, we found that Dvl2 binds to Nkd1 in a partially overlapping region with its β-catenin-binding site (Fig. 5D, S2). Previously we demonstrated that Axin1 binds to Nkd1 via the C-terminal poly histidine tail on Nkd1 (Fig. 5D) [28]. Our current analysis demonstrates that this region is not required for binding to either Dvl2 or β-catenin suggesting that Axin1 binding to Nkd1 is not a requirement for Nkd1 binding to Dvl2 or β-catenin.

These mapping results support previous findings. Using just the EF-hand, Wharton et al., found this region was sufficient to bind Dvl [10], while Yan et al., found Dvl still bound Nkd in the absence of the EF-hand [22] and we previously found that Dvl binds a domain in the C-terminal region on Nkd2 [20]. Therefore, Dvl2 (and β-catenin) bind to regions encompassing the EF-hand and a separate domain C-terminal to the conserved EF-hand domain, which includes the NHR2 domain. Fine interaction mapping of these regions is currently underway, which will determine more precisely where Dvl2 and β-catenin bind Nkd1 and if these two proteins bind to the same or juxtaposed regions of Nkd1. Thus, we conclude that the Nkd1-β-catenin interaction is conserved between zebrafish and mammals. Moreover, our data also suggests that Dvl2 and β-catenin likely compete for binding to Nkd1.

## Discussion

Activation of canonical Wnt signaling is well characterized and is considered to proceed through the formation of Wnt-induced signalosomes [47], [48]. What is less well understood is how this pathway controls its own activity to limit the amount of Wnt signaling. Here we show that Nkd1, a Wnt-inducible negative-feedback regulator limits Wnt signaling by preventing nuclear accumulation of β-catenin. This work furthers our understanding of the role Dvl plays in this process. We have found that Nkd1 interacts with both a slow and fast migrating form of Dvl2 correlating with phosphorylated and unphosphorylated forms of Dvl2, respectively [40], [41], [42], [43], [49]. In the presence of Nkd1, exogenous Dvl also becomes enriched at the plasma membrane. Thus, it appears that plasma membrane localization of Nkd1, via myristoylation, is required for its association with a slower migrating (phosphorylated) form of Dvl. This is supported by our observations that, in the absence of myristoylation, Nkd1^G2A^ interacts preferentially with a faster migrating form of Dvl and both remain uniformly distributed within the cytoplasm. Alternatively, the binding of Nkd1, but not Nkd1^G2A^, to Dvl, may lead to Dvl phosphorylation, but this does not account for the observation that Nkd1 interacts with both a phosphorylated and unphosphorylated form of Dvl.

Thus far, this data fits well with Nkd1 antagonism occuring at the level of Dvl, simply inhibiting Dvl in the signalosome. However, this model cannot account for the lack of effect of Nkd1 on the levels of cytoplasmic β-catenin. While Axin can effectively reduce the levels of cytoplasmic β-catenin induced by Wnt8, Nkd1 cannot. In fact, we often observed slight (but not significant) increases in cytoplasmic β-catenin in the presence of Nkd1, without Wnt stimulation. Further, we observed that Nkd1 physically interacts with β-catenin and that the strength of this interaction is dependent on an intact myristoylation sequence in Nkd1. This suggests that Nkd1 needs to associate with the plasma membrane before it can interact with β-catenin. Also, we have determined that β-catenin and Dvl overlap in Nkd1 binding domains, that they likely compete for binding to Nkd1, and that Wnt signaling may promote binding between Nkd1 and β-catenin. Finally, our observation that Nkd1 inhibits accumulation of nuclear β-catenin suggests that this effect of Nkd1 takes place in the cytoplasm.

Recently, Axin1 and Axin2 were also found to interact with Nkd1 through the C-terminal poly-histidine tail of Nkd1 [28], but it is not yet clear if this interaction is occurring in the cytoplasm or at the plasma membrane or both. Axin has been found to form both plasma membrane localized and intracellular puncta, but these two domains act opposite to one another with respect to Wnt signaling. Wnt ligands induce the formation of plasma membrane-localized Axin clusters which also contain Dvl and LRP5/6 proteins forming a Wnt signalosome [48], while cytoplasmic Axin clusters co-localize with APC and β-catenin and constitute the constitutively active destruction complex[50]. Also, it is well documented that these clusters are aggregates of Wnt signaling components and not true vesicles [36], [48]. Thus, as Nkd1 binds to Axin [28], Dvl [10], [22], [23], [39] (this study) and β-catenin [28] (this study), it is likely that the observed Nkd1 puncta are also part of a Wnt signalosome and/or destruction complex, although this requires formal testing.

In Drosophila, Nkd was also tested for interactions with Axin and β-catenin by yeast-2-hybrid but no interaction was detected [23]. Since we found that membrane localization of Nkd1 is critical for its interaction with β-catenin, it is possible that the yeast-2-hybrid environment could not recapitulate a possible membrane requirement for Drosophila Nkd to interact with β-catenin. This is supported by the finding that fly Nkd function is also dependent on its interaction with the membrane [27]. Alternatively, the interaction between Nkd/Nkd1 and β-catenin requires an intermediate protein, which would be available in vivo, but not available in a yeast-2-hybrid assay.

Myristoylation of Nkd1 is critical for its activity and to understand this further, we generated Nkd1 chimeras to target Nkd1 to the membrane in the absence of myristoylation to determine if these chimera's could recapitulate wild type Nkd1 activity. Tagging Nkd1 with alternate membrane localization motifs (N- or C-terminal Plekstrin Homology domain, a N-terminal signal sequence with transmembrane motif, and a C-terminal CAAX domain) could not recapitulate Nkd1 activity and in all cases could not localize, or only weakly localize, to the plasma membrane (TVR, LS-K, RJC unpublished data). This suggests that myristoylation is specifically effective at bringing Nkd1 to the membrane.

The exact mechanism of Nkd1 action is still unknown, but may involve the nuclear import machinery. In *Drosophila* it was found that Nkd Cuticle binds to Importin alpha3 via its ARM repeats and that this region in Nkd is critical for its function [24]. Interestingly, Importin alpha3 shares significant homology to β-catenin specifically in the ARM-repeats and we are currently testing the hypothesis that Nkd1 and β-catenin interact through the β-catenin ARM repeats. Fly Nkd also contains nuclear localization sequences and is observed in the nucleus. While these nuclear localization sequences are conserved between fly and mosquito Nkd proteins, they are not conserved in vertebrate Nkds. Furthermore, suppression of nuclear export in *Drosophila* cells did not result in nuclear accumulation of *Drosophila* Nkd [26], arguing against a role for Nkd in the nucleus. While we can not rule out a role for vertebrate Nkd1 in the nucleus, the absence of conserved nuclear localization sequences and the presence of a myristoyl moiety suggests that, at least in zebafish blastula cells, Nkd1 is unlikely to have a significant role in the nucleus.

Our observations that Nkd1 has a more dramatic effect on ectopic Wnt signaling compared with endogenous Wnt signaling appears to reflect an evolutionarily conserved phenomenon. In fly, Nkd is only required during early segmentation of the Drosophila embryo and not during other aspects of development involving Wg signaling [51]. In vertebrates knockout of both Nkd1 and Nkd2 has no overt phenotype [52]. We have also knocked down zebrafish Nkd1 and Nkd2 and observed modest, but significant, changes in Wnt signaling [9], and overexpression of Nkd, Nkd1 or Nkd2 by itself does not have a dramatic or consistent phenotype in wild-type embryos [9], [51]. Only under compromised Wnt signaling conditions do we observe Nkd1 function, either by overexpression of Wnt or by genetic manipulation that results in reduced or ectopic Wnt signaling [9] (this study and TVR, LS-K, RJC unpublished). This is entirely consistent with observations in Drosophila [23], [51] and suggests that there is a threshold of Wg/Wnt signaling upon which Nkd functions. We predict that this threshold would be lower in a Wg/Wnt sensitized background. This could explain the subtle loss of function phenotype in the Nkd1/Nkd2 double knockout mouse if this threshold is never perturbed under normal physiological conditions, or why there are not additional *nkd*-/- phenotypes in the developing Drosophila embryo.

In conclusion, we have demonstrated that in zebrafish blastula cells, Nkd1 prevents nuclear accumulation of β-catenin and that this function of Nkd1 is dependent on its plasma membrane localization via myristoylation. Furthermore, we also found Nkd1 to interact with β-catenin and that Dvl2 and β-catenin may compete for binding to Nkd1. The functional significance of Nkd1 interacting with β-catenin, Dvl2 and Axin is currently under investigation.

## Materials and Methods

### Quantitative RT-PCR

Total RNA from embryos was prepared using Trizol (Gibco). cDNA was synthesized with Superscript II (Invitrogen). Quantitative RTPCR (qRT-PCR) was performed using IQ Syber green (BioRad). Three replicates were performed for each experiment. Sequences of primers were as follows. β-Actin Forward: 5′-ATGCCCCTCGTGCTGTTTT-3′; β-Actin Reverse: 5′-TCTGTCCCATGCCAACCAT-3′; Nkd1-5′UTR Forward 5′- AAGCCGCGCGCTCTTC-3′; Nkd1-5′UTR Reverse 5′- GAAACATCAGAAACAAAAAACAATCC-3′; Axin2 Forward: 5′-ATGCCCCTCGTGCTGTTTT-3′; Axin2 Reverse 5′-TCTGTCCCATGCCAACCAT-3′. Absolute quantification was determined using a standard curve generated by β-*actin*. Relative quantitative RT-PCR (qRT-PCR) was determined by normalizing to uninjected.

### Constructs

Nkd1^G2A^ constructs were generated by site directed mutagenesis. Constructs were cloned into the ClaI site of pCS2+myc or pCS2+GFP. Mutagenesis primers are as follows Nkd1^G2A^ For 5′- GCAGGATCCCATCGATGATGGCTAAACTTCATTCC-3′; Nkd1^G2A^ Rev 5′- GGAATGAAGTTTAGCCATCATCGATGGGATCCTGC-3′; Orientation and sequence integrity were confirmed by sequencing. pCS2+Axin1 was kindly provided by Toshio Hirano [33]. pCS2+Wnt8 was kindly provided by Randall Moon [31], [53].

### mRNA injections

Capped mRNA was synthesized and purified using Ambion's mMessage mMachine kit following their protocol. mRNA was diluted in RNase free water, and its quantity and quality was analyzed by spectrophotometer and RNase free gel analysis. For all of the experiments, except where noted, 800 pg of *nkd1* ^myc^, *nkd1^GFP^; nkd1^N-GFP^; or* 1200 pg *nkd1^G2A-myc^ or nkd1^G2A-GFP^;* 25 pg *wnt8*; 100 pg *dsh2-^HA^*; 400 pg *axin1* in 1% phenol red were pressure injected into the yolk cell of one- to two-cell-stage embryos. For immunohistochemistry on nuclear β-catenin, a mixture of 25 pg *wnt8* and 100 pg of *nkd1^GFP^* or *nkd1^N-GFP^* was pressure injected into one of four blastomeres.

### Fractionations

Ten embryos were harvested at dome stage (4.3 hpf), mechanically dechorionated and deyolked in 0.3X Danieau. Deyolked animal caps were lysed by trituration in TKM buffer (50 mM Tris-HCl 7.5; 25 mM KCl; 5 mM MgCl_2_; 1 mM EGTA; 0.02% Na Azide) containing Complete® Protease Inhibitor (Roche). Lysates were centrifuged at 1000g for 5 min at 4°C to pellet nuclei, large membrane fragments and whole cells. The resulting lysate was further fractionated into crude plasma membrane and cytoplasmic fractions by centrifugation at 100,000g for 1 hr at 4°C.

### Immunoprecipitations

Cells were lysed through an 18 guage needle in modified Rubenfelds buffer [54] (modification: 0.3% NP-40 replaced 1% Triton-X) containing Complete Protease Inhibitior® (Roche). Primary antibodies consisted of (monoclonal anti-β-catenin, Sigma; monoclonal anti-myc 9E10, Vanderbilt Antibody Core; polyclonal anti-Dvl2 generated against the zebrafish Dvl2 peptide HSSGSTRSDGEKKRRGPKSVSE, corresponding to amino acids 573–592 and conjugated on the C-terminus with a Cysteine (Covance).

### Western Analysis

Monoclonal anti-myc 9E10 1∶2000; monoclonal anti-β-catenin, 1∶1000; polyclonal anti-GFP (Torrey Pines) 1∶1000, polyclonal anti-pan-cadherin (Abcam) 1∶1000, monoclonal anti-actin (Sigma) 1∶2000; polyclonal anti-Dvl2 1∶5000; anti-HA (Vanderbilt Antibody Core) 1∶1000.

### Immunohistochemistry

Primary antibodies: mouse anti-myc 1∶500; Rat anti-HA 1∶200; Rabbit anti-zDvl2 1∶1000 or 1∶5000; mouse anti-β-catenin 1∶250). Secondary antibodies: Cy2 Donkey anti-Rat 1∶200; Cy3 Goat anti-mouse 1∶200; Cy3 Goat anti-Rabbit 1∶200; Cy2 Goat anti-Rabbit 1∶200; AlexaFluor 594 1∶200; syto59 1∶10,000.

### Nuclear β-catenin

Whole mount immunhistochemistry: Nuclear β-catenin staining was carried out using the Vectastain Elite ABC kit (Vectastain Laboratories) along with Avidin D (blocking kit, Vectastain Laboratories) and ImmPact DAB (Vectastain Laboratories).

Immunohistochemistry: To quantify the changes in nuclear β-catenin, 6 cells Nkd1^GFP^ positive and 6 juxtaposted GFP negative cells, and 10 cells for Nkd1^N-GFP^ positive and juxtaposed GFP negative cells were analyzed. Grey scales for Nuclear Hoechst and nuclear β-catenin where calculated using Image J. They grey levels of signal intensity were measured across the cell for both the Hoechst staining and the β-catenin staining. For each cell, the average level of β-catenin in the nuclei was normalized to its Hoechst staining. The adjusted averages were then plotted and compared by students T-test.

### Nkd1 Interaction Mapping

Immunoprecipitation (IP) and immunoblotting (IB) were carried out using an anti-flag antibody (Sigma #F3165; 1∶1000 IP, 1∶3000 IB). LUMIER experiments were performed as previously described [28], [55].

## Supporting Information

Figure S1 **Nkd1 inhibits nuclear β-catenin.** (A) Whole embryo analysis of nuclear β-catenin staining as per Fig. 4.) (B) Immunohistochemistry of Nkd1^GFP^ and Nkd1^N-GFP^ co-injections with *wnt8*. Injections are labeled along the left side, staining is identified along the top.
(TIF)

Figure S2 **Dvl2 and β-catenin bind to similar domains on Nkd1.** HEK293T cells were transfected with luciferase-tagged β-catenin (left panel) or Dvl2 (right panel) and the indicated 3XFlag-Nkd1 constructs. Cell lysates were subject to anti-Flag immunoprecipitation and the presence of β-catenin or Dvl2 was assessed by luciferase assay. Luciferase activity of the immunoprecipitate was normalised to that of the total cell lysate (left panel) or plotted separately (right panel). Data are the average of at least 4 independent experiments expressed as % wild type +/− SEM (left panel) or as the mean of 2 samples +/− standard deviation of a representative experiment (right panel).
(TIF)
