# Supplementary figures and images for: Naked1 Antagonizes Wnt Signaling by Preventing Nuclear Accumulation of β-Catenin
Source: PLoS One. 2011 Apr 7;6(4):e18650. doi: 10.1371/journal.pone.0018650 (PMC3072412; doi:10.1371/journal.pone.0018650)

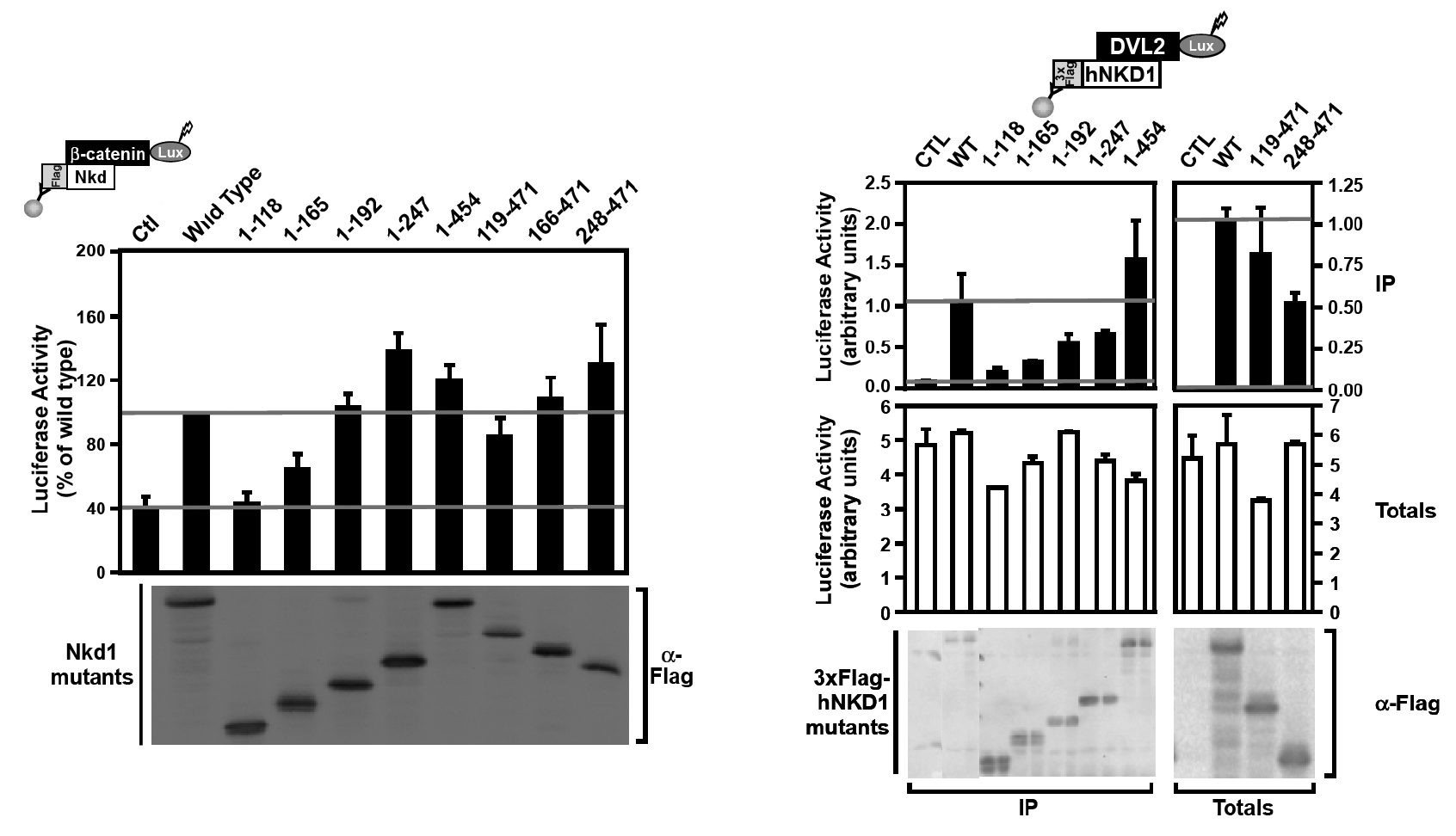

Supplement: Figure S2 — Dvl2 and β-catenin bind to similar domains on Nkd1. HEK293T cells were transfected with luciferase-tagged β-catenin (left panel) or Dvl2 (right panel) and the indicated 3XFlag-Nkd1 constructs. Cell lysates were subject to anti-Flag immunoprecipitation and the presence of β-catenin or Dvl2 was assessed by luciferase assay. Luciferase activity of the immunoprecipitate was normalised to that of the total cell lysate (left panel) or plotted separately (right panel). Data are the average of at least 4 independent experiments expressed as % wild type +/− SEM (left panel) or as the mean of 2 samples +/− standard deviation of a representative experiment (right panel). (TIF) [file pone.0018650.s002.tif]
